# Adolescent mental health and academic performance: determining evidence-based associations and informing approaches to support in educational settings

**DOI:** 10.1038/s41390-024-03098-3

**Published:** 2024-02-27

**Authors:** Xzania Lee, Anya Griffin, Maya I. Ragavan, Mona Patel

**Affiliations:** 1 Children’s Hospital Los Angeles, Los Angeles, CA USA; 2 Keck School of Medicine at the University of Southern California, Los Angeles, CA USA; 3 Division of General Academic Pediatrics, University of Pittsburgh and UPMC Children’s Hospital of Pittsburgh, Pittsburgh, PA USA

In 2021, the American Academy of Pediatrics, the American Academy of Child and Adolescent Psychiatry and the Children’s Hospital Association (CHA) declared a “National State of Emergency in Children’s Mental Health.”^1^ The statement identified how the pandemic exacerbated the already worsening mental health problem among US youth due to the compounding challenges faced by youth and acknowledged the significant impact of this mental health crisis on youth. This declaration made a call for schools, policymakers, and advocates for children and adolescents to prioritize and focus on pediatric mental health.

Adolescent mental health and academic performance are intricately linked aspects of development, each influencing and being influenced by the other. The recognition of this bidirectional association has sparked considerable interest within the research community, prompting an investigation into the nuanced dynamics between mental health and educational outcomes during the formative adolescent years. Numerous studies have explored the connection and influence between mental health and academic performance, and further acknowledge that the multifaceted interconnectedness of mental health and academic performance require a holistic view.^2–4^ Researchers have identified that higher academic aspirations are associated with better mental health outcomes and that socioemotional well-being is needed for academic thriving.^5,6^ Furthermore, Yu and associates described how interpersonal relationships are positively correlated with academic performance, especially student-peer relationships, which had more influence than the parent-student or teacher-student relationship on academic achievement.^7,8^ Finally, impacts of social determinants of health have been shown to exert profound influences on both mental health and academic outcomes further emphasizing the need to consider the broader ecological context in which adolescents develop and the importance of considering a socioecological model, suggesting that factors such as family, school, and community environments play pivotal roles in shaping both mental health and academic outcomes.^6,9^

In this article by Monzonis-Carda and associates, the authors explore the bidirectional longitudinal association between the dual-factor model of mental health and academic performance in adolescents. The dual-factor model of mental health, in contrast to traditional models of mental health which focus on psychopathological symptoms, integrates mental health wellbeing and psychopathology into a mental health continuum.^10^ The authors hypothesize that a bidirectional association between academic performance and adolescent mental health would be present in their sample of 266 secondary school students from Spain. They assessed mental health through the Spanish language Behavior Assessment System for Children and Adolescents (BASC-S3) and examined grade point average, and academic performance based on the Test of Educational Abilities. They then employed a cross-lagged modeling approach to analyze the bidirectional association over 2 years. The key findings suggested that higher academic performance at baseline was associated with better mental health over time, but better mental health was not associated with academic performance. Therefore, the association was not bidirectional as expected. Based on these findings, the authors posit academic performance may be a predictor of adolescents’ mental health status; and conversely, mental health may not be a predictor of adolescents’ academic performance. They offered school-based recommendations for the promotion of good mental health practices for students with low academic performance and supported future policy and health and educational professionals to promote adolescent mental health wellbeing. Overall, the article underscores the importance of considering academic performance as a target for interventions to promote adolescents’ mental health. It suggests that focusing on reducing school pressure and establishing personalized academic goals could contribute to better psychological well-being.

While the article provides some important insights into the association between mental health and academic performance in adolescents, some limitations were noted. While there is some limited adjustment for socioeconomic status, the article lacks a comprehensive exploration of social determinants of health and impacts of adverse childhood events (ACES), such as cultural background, and other important social and familial dynamics. These factors play a pivotal role in shaping an adolescent’s mental health and academic performance and may result in an oversimplified understanding of the complex interplay between mental health and academic outcomes. The study further focuses on academic grades and “abilities” as indicators of academic performance. Academic success is multifaceted and includes factors like motivation, engagement, and teacher-student relationships, and a more nuanced exploration of these components could provide a richer understanding of the relationship between mental health and academic outcomes. The study authors reviewed limitations that require further investigation including the use of BASC-S3 as the primary self-reported measure of adolescent mental health. Depending on individual developmental level of insight and situational context, adolescents are often unreliable and inaccurate reporters of their functioning, and adolescents in clinical populations tend to overreport symptoms and provide inaccurate information regarding their functioning on the BASC-S3.^11,12^ Incorporating objective measures or multi-method assessments and the inclusion of multi-rater methods (i.e., teachers, caregivers, etc.) may provide a more detailed picture of the student’s true socioemotional functioning through the provision of differing perspectives of each student’s functioning.^13^ The study’s authors also acknowledge a relatively small sample size, homogeneity of the study population, and short study length to determine longitudinal outcomes may further limit generalizability to other populations. Lack of testing for sex assigned at birth and self-identified gender effects, and not integrating broader social determinant impact upon adolescent mental health may result in misguided or ineffective approaches to promoting mental health in adolescents. Further, previous research and psychological assessment literature have indicated the significant impact of social determinants of health and ACES on youth academic achievement and behavioral health outcomes. Students with elevated social risk, including ACES, are often at increased risk for mental health and academic achievement deterioration.^14^ This supports the need for school leaders and policymakers to continue to focus efforts on maximizing the recognition of these factors for youth and promote the implementation of programs to address roots of social risk and integration of socioemotional mental health supports in academic institutions.^15^ Due to the interconnectedness of mental wellness and academic success, addressing aspects of mental health functioning within the school setting will equip students with the essential skills to navigate challenges, manage stress, and build resilience. By bolstering emotional, behavioral, and social skills, students are primed to engage in learning, establish positive relationships with peers and teachers, and cope with the pressures of academic stress and daily life hassles.^16^ A structured educational tier one (i.e., general education curriculum) mental health intervention will assist students with stress reduction, and behavior management, improve executive functioning skills, and establish a scholastic environment conducive to effective knowledge consumption and academic performance.^17^ Incorporating evidence-based practices to support student emotional wellness holistically nurtures the development of students and provides a foundation for lifelong well-being and academic excellence. While this article contributes to the understanding of the association between mental health and academic performance, it also highlights the need for future exploration of factors that influence the causality between adolescent mental health and academic performance and further informs the recommendation to have mental health interventions and social-emotional learning curriculums in educational settings.

The 2021 joint declaration of the “National State of Emergency in Children’s Mental Health” catalyzed federal, state, and local awareness of evolving needs in pediatric mental health in the United States of America. While there has been increasing bipartisan support and focus for mental health funding at all levels of government, appropriate allocation of such funding to support identifying factors that impact pediatric mental health and using a data-driven approach to effective programming is critical. An example of more recent federally supported funding programs for child mental health includes the Health Resources & Services Administration (HRSA) funded Pediatric Mental Health Care Access (PMHCA) program, which has seen increased funding from 2018 through 2022, with an additional 80 million dollars added by the Bipartisan Safer Communities Act. (https://mchb.hrsa.gov/programs-impact/programs/pediatric-mental-health-care-access) Currently, under-resourced and under-reimbursed health systems fraught with post-pandemic short staffing and pre-pandemic existing behavioral health access challenges pose continued roadblocks to access. Pediatric policy recommendations to aid with improving meaningful pediatric mental health access include:

- Increased funding and support for access to meaningful mental health resources in the community and schools
- Integrated behavioral health delivery models within primary care and specialty care will be critical in enhancing access to care.
- Increase the behavioral health workforce, training programs for primary care pediatricians and pediatric psychologists are needed, as the number of child psychiatrists and pediatric psychologists is currently not sufficient to meet demand.
- Innovative and integrative team-based models including non-traditional licensed and non-licensed behavioral health support teams, including community health work may allow further access and a more impactful peer-to-peer support structure.

Behavioral health reimbursement shifts may ultimately be required to build infrastructure to address the current critical socio-emotional needs of our youth. Ultimately, research informing a more comprehensive perspective, including health-related social needs and ACES will be essential for advancing the field with evidence-based mental health interventions for youth.
